# Clinical Tests of New Drugs

**Published:** 1916-01-22

**Authors:** 


### CLINICAL TESTS OF NEW DRUGS.

Any discussion bearing on treatment naturally engages the interest of members of the medical profession, and particularly of those who are actively engaged in family practice. From the point of view of the patient the efficiency of the practitioner is judged by the success with which he can afford relief from the sufferings or other inconveniences produced by illness. Scientific knowledge and attainments may be all very well, but the sick man wants to be cured, and it is for this purpose he consults his medical adviser. For the majority, too, the cure must certainly include something in the shape of drug administration. There may be other directions and suggestions, but the scheme will usually be considered incomplete unless it comprises a definite something which may be regarded as a remedy. Not yet has the public learned to banish all idea of the mysterious from the art and practice of medicine. Possibly this expression of faith in the capacities of drugs is excessive, but it exists, and has to be taken note of in the conduct of affairs. As opposed to it is an incredulity which often finds expression in medical circles, and which would have been classed as rank heresy by practitioners of an older generation. The truth probably lies somewhere between these two extremes. If the public is credulous and expects too much, the unbeliever is at least often one who has not learned to handle his tools. "Drugs, I suppose, are no use," once remarked a distinguished surgeon in consultation with an equally distinguished physician. "Not the slightest," was the somewhat unexpected reply, "unless you know how to use them." There is a moral in the incident which ought not to be lost by the superior person who professes to have "no faith in drugs."

At the same time that incredulity is announced in certain professional quarters there is a somewhat pathetic hopefulness often cultivated towards all new therapeutic proposals. The manufacturing chemist and the advertiser provide a rich supply of such proposals, and at times both doctors and patients are debtors to such enterprises. Yet everyone will admit that the great majority of these schemes, after a more or less brief career, fall into absolute oblivion. This fate attends not a few which from a commercial point of view have been very successful. The long arm of coincidence, a readiness to see good results, and imperfect observations, have often combined to support therapeutic claims which time has shown to be baseless. Obviously it is much to be desired that these unwarranted professions should be terminated as promptly as possible. Yet such an end is not easy to attain. Commercial interests keep them in the forefront, and the influences just mentioned often secure for them what seem to be impressive testimonials. On the other hand, it is no one's interest to prick the bubble.

The practitioner who finds the new agent valueless is not tempted to rush into print with the experience, and thus the assertions on the other side have free course. The publication of a series of careful observations conducted under hospital conditions and announcing the real value of some of these claims would be a boon both to the profession and the public. The natural inclination is to record therapeutic triumphs, but therapeutic failures also have their lesson. Such failures are not easily announced by the private practitioner, and to be of much value they must rest on observations conducted in circumstances favourable to strict accuracy and to impartial judgment. Our hospitals could in this direction do more than they accomplish at present, and might thus contribute still further to the education of medical opinion on various new therapeutic proposals.

